# Effects of Exogenous Melatonin on Life History Traits and Cold Tolerance of *Leguminivora glycinivorella* (Lepidoptera: Tortricidae)

**DOI:** 10.3390/biology15100750

**Published:** 2026-05-09

**Authors:** Shiyu Zhu, Yichang Xing, Yuxin Zhou, Shusen Shi, Yu Gao

**Affiliations:** 1 College of Plant Protection, Jilin Agricultural University, Changchun 130118, China; zhushiyu@jlau.edu.cn (S.Z.);; 2 Key Laboratory of Soybean Disease and Pest Control, Ministry of Agriculture and Rural Affairs, Changchun 130118, China

**Keywords:** cold tolerance, exogenous melatonin, fecundity, growth and development, *Leguminivora glycinivorella*, nutrition metabolism

## Abstract

The chemical control of *Leguminivora glycinivorella* (Mats.) (Lepidoptera: Tortricidae) has been a major threat to the soybean industry in China over the years. Therefore, we need to develop green, safe, and environmentally friendly alternatives for pest control. Amine hormones, such as melatonin, represent an adjunct strategy for the green control of *L. glycinivorella*. We aimed to investigate the effects of exogenous melatonin on the development, survival, reproduction, nutrient accumulation, and cold tolerance of *L. glycinivorella* and to provide insights into the role of melatonin in insect adaptive regulation. Newly hatched *L. glycinivorella* larvae were fed on soybean pods immersed in solutions with different concentrations of exogenous melatonin. The developmental duration of larvae and pupae, survival rate, pupation rate, adult eclosion rate, body weight and length of larvae and pupae, nutrient composition of mature larvae, adult reproductive parameters, and supercooling and freezing points were measured. With increasing melatonin concentrations, the developmental durations of larvae and pupae were significantly prolonged, while the larval survival rate, pupation rate, and adult eclosion rate significantly decreased. The body weight and length of both larvae and pupae declined with increasing melatonin concentrations, reaching the lowest values in the 200 mg/L group. Melatonin treatment significantly reduced protein and lipid contents in mature larvae but significantly increased glycogen content. Reproductive parameters, including pre-oviposition period, oviposition period, fecundity, and female adult longevity, all decreased significantly with increasing melatonin concentrations. Additionally, melatonin treatment significantly lowered the supercooling point and freezing point of mature larvae. Exogenous melatonin significantly inhibits the growth, development, survival, reproduction, and nutrient metabolism of *L. glycinivorella*, yet enhances its low-temperature tolerance, suggesting that melatonin may serve as a potential tool for population management of *L. glycinivorella* through its dual regulatory role in insect physiology.

## Simple Summary

The soybean pod borer, *Leguminivora glycinivorella* (Mats.) (Lepidoptera: Tortricidae), is a major destructive pest of soybean. Due to the numerous adverse effects of chemical control, there is an urgent demand for green and safe strategies for managing this pest. In this study, newly hatched larvae of *L. glycinivorella* were reared on soybean pods soaked in different concentrations of melatonin, and their growth, development, reproduction, nutrient composition, and cold tolerance were investigated. The results showed that higher melatonin concentrations significantly prolonged larval and pupal development and markedly reduced the survival rate, pupation rate, adult eclosion rate, body size, body weight, fecundity, and reserves of nutrients such as protein and fat. Additionally, melatonin treatment lowered the supercooling and freezing points, enhancing the cold tolerance of the larvae. This study demonstrates that high concentrations of melatonin can inhibit the growth and reproduction of *L. glycinivorella* while simultaneously improving its low-temperature tolerance, providing a scientific basis for its potential application in pest management.

## 1. Introduction

Soybean (*Glycine max* (L.) Merr.) is one of the most economically important crops in the world [1]. In China, soybeans are a major raw material for edible oil and a wide range of soybean-based products (e.g., soymilk and tofu) [2]. The soybean pod borer, *Leguminivora glycinivorella* Mats. (Lepidoptera: Tortricidae), is the most serious borer pest of soybean [3,4]. Its larvae feed on pods and developing seeds. This feeding not only reduces soybean yield but also severely impairs grain quality. The simultaneous decline in yield and quality results in substantial economic losses [5]. In years of severe outbreaks, the pod damage rate can reach 50–70%, which leads to a 20–40% reduction in soybean yield [6]. *L. glycinivorella* has been described as probably the most serious pest of more than 70 insect species associated with soybeans [7]. It is also listed as a quarantine pest in the United States [8]. Currently, chemical control remains the primary method for the rapid suppression of *L. glycinivorella* outbreaks [9,10].

Previous studies have shown that the use of conventional chemical insecticides in global agricultural production imposes strong selection pressure on pest populations and accelerates the evolution of insecticide resistance [11]. Many soybean pests have already developed resistance to commonly used insecticides. For example, field populations of *Rachiplusia nu* (Guenée) in Brazil exhibit resistance to the insecticides flubendiamide and bifenthrin that is 11–31 times higher than in susceptible populations presenting low to moderate levels of resistance [12]. In addition, Brazilian lepidopteran pests show highly variable sensitivity to indoxacarb, spinetoram, and flubendiamide [13], while USA studies confirm widespread pyrethroid resistance in the soybean aphid (*Aphis glycines* Matsumura) [14]. The sensitivity of *L. glycinivorella* to commonly used insecticides, such as diamides and pyrethroids, has also declined significantly [15]. Therefore, it is essential to explore new control technologies and management strategies for *L. glycinivorella* and to develop environmentally friendly pest control approaches [16]. This need has both strong scientific significance and urgent practical relevance. In this context, various non-chemical control methods have attracted attention, including natural enemy utilization (e.g., *Diadegma hiraii* (Kusigemati) [15]), sex pheromone-based control [4], and microbial pesticides (e.g., *Beauveria bassiana* (Bals.-Criv.) Vuill.) [17]. In addition, melatonin and related amine hormones show great promise as supplementary pest control agents; research in this field is essential for developing eco-friendly alternative technologies.

Melatonin is widely distributed in many categories of organisms, such as vertebrates, invertebrates, plants, and unicellular algae [18,19,20]. Since the first detection of melatonin in the compound eye of *Locusta migratoria* L. [21], it has been identified in several insect species [22,23,24,25,26,27]. Melatonin acts as an important physiological signal that conveys photoperiod information and helps synchronize endogenous rhythms with environmental cycles [28]. It also functions as an antioxidant and a growth regulator. In insects, melatonin participates in several physiological processes, including metabolism, immune activity, embryonic development, molting, reproductive behavior, and stress responses [29,30,31]. Studies have shown that exogenous melatonin significantly inhibits the growth and reproduction of many insect pests. There are few studies on exogenous melatonin in Lepidoptera, such as *Spodoptera frugiperda* (J.E. Smith), in which exogenous melatonin, especially at high concentrations, significantly inhibits its growth and development by prolonging larval duration, reducing body size and weight, shortening adult lifespan, and markedly suppressing female reproduction via lowering fecundity and slowing ovarian development [32,33,34,35,36]. Exogenous melatonin can prolong the pest developmental period, inhibit feeding and growth, and reduce reproductive capacity and survival, with these effects intensifying markedly at higher melatonin concentrations. Melatonin may contribute to pest control through two main mechanisms. It can directly inhibit pest growth and reproduction, and it can indirectly enhance the resistance of host plants. Therefore, melatonin shows strong potential as an environmentally friendly tool for pest management.

However, few comprehensive, systematic studies have elucidated the regulatory effects of exogenous melatonin on the growth, development, reproduction, nutrient composition, and cold tolerance of *L. glycinivorella*. To bridge this critical knowledge gap, the present study systematically assessed the effects of exogenous melatonin on the life history traits, nutrient reserves, and cold tolerance of *L. glycinivorella*, thereby providing a scientific basis for the potential application of melatonin in the sustainable management of this pest. Accordingly, this study systematically evaluated the effects of exogenous melatonin on the life history traits, nutrient reserves, and cold tolerance of *L. glycinivorella*, aiming to provide scientific support for the potential application of melatonin in the sustainable management of this pest.

## 2. Materials and Methods

### 2.1. Method of Insect Collection and Rearing

Adult *L. glycinivorella* were collected in August 2023 from Changchun City, Jilin Province (43°48′36″ N, 125°24′10″ E). Eggs laid by these adults were used as the insect source for this experiment. During the peak eclosion period, adults were captured daily before sunset using net traps from soybean field plots that had not received any pesticide application for over 15 consecutive years, with the collected individuals serving as the experimental insect source. Collected adults were transferred to a high-transparency plastic insect rearing jar (12 cm in diameter, 15 cm in height), each equipped with 3 high-density mesh-covered ventilation holes (3.5 cm in diameter) to ensure stable air circulation and to prevent test insect escape. The insect rearing containers were also supplied with 10% honey water as a nutrient source and placed in an artificial climate incubator (GXZ380B, Jiangnan Instrument Factory, Ningbo, China) under the conditions of 25 ± 1 °C, 70% ± 10% relative humidity, and a photoperiod of 16:8 h (L:D) [37]. Soybean pods were replaced every 12 h. Soybean pods bearing newly laid eggs of *L. glycinivorella* were wrapped at the base with moistened absorbent cotton for moisture retention and then placed in glass Petri dishes lined with moist filter paper for egg hatching. For the test, fresh eggs laid daily by mated adult moths on soybean pods were used as the initial source of the experimental insect population. The soybean cultivar ‘Jiyu 303’ was used as the oviposition substrate; the soybean plants were cultivated at the experimental field. No chemical insecticides were sprayed throughout the entire soybean growth season so as to eliminate the interference of pesticides on the experimental insect population.

### 2.2. Methods for Measuring Growth, Development, and Reproductive Parameters

Melatonin treatment was performed via a dietary feeding method, which was modified based on the protocols described in previous studies (Yamano et al. [38]; Zhang et al. [26]). Melatonin (purity ≥ 99.00%, Bioengineering Co., Shanghai, China; molecular formula: C_13_H_16_N_2_O_2_) was dissolved in deionized water. In our study, solutions with concentrations of 0.2, 2, 20, and 200 mg/L were prepared and stored in a refrigerator. Our preliminary experiments verified that exogenous melatonin significantly affected the developmental duration, body weight, and body length of mature 5th instar *L. glycinivorella* larvae and preliminarily clarified the effective concentration range of melatonin for this insect. Thus, we set the formal melatonin treatment concentrations using a 10-fold linear increasing gradient, which was in line with the classic concentration setting protocol of exogenous melatonin in representative studies on *Spodoptera frugiperda* [26]. Therefore, the four concentrations of exogenous melatonin mentioned above were used in this study. Larvae were reared under a photoperiod of 16:8 h (L:D), corresponding to a non-diapausing environment for *L. glycinivorella*, as defined by diapause criteria proposed by Li et al. [37]. The climate chamber (GXZ380B, Jiangnan Instrument Factory, Ningbo, China) was maintained at 21 ± 1 °C with a relative humidity of 85 ± 5%. CK (control check) was the solvent blank control group, treated with 0 mg/L exogenous melatonin in clean water (the sole solvent for all melatonin working solutions in this study), with rearing and handling conditions identical to all melatonin treatment groups.

Newly hatched *L. glycinivorella* larvae have an obligate non-pod-switching biological trait: they feed only within the initial bored pod until mature pod exit, so only the initial inoculation pods were treated with melatonin soaking in this study. Initial pods for treatment and control groups were from the same soybean cultivar, identical pod-setting stage, and uniform size, plumpness, and health status, with all procedures fully synchronized except for melatonin treatment. Pre-experiments confirmed that over 85% of larvae completed full larval development solely on the initial treated pods, with only a tiny number of samples needing replacement with identical standard untreated pods under extreme conditions (e.g., severe mold).

Fresh soybean pods of consistent size and maturity were soaked in freshly prepared melatonin solutions at the set concentrations for 12 h. After soaking, newly hatched *L. glycinivorella* larvae were individually transferred to the surface of the treated pods using a soft, fine brush to avoid mechanical damage; each inoculated pod was wrapped in a small piece of absorbent paper towel and placed in a 10 mL centrifuge tube with a perforated lid to ensure adequate ventilation throughout the individual larval rearing period.

The pod depodding status of *L. glycinivorella* was observed every 12 h, and its body weight and body length were measured on the second day following pod depodding. Larvae were observed daily to record the number entering pupation, and the pupation rate of depodded larvae was calculated. The body weight and body length of the pupae were measured first. Adult eclosion events were then recorded daily at a fixed time point, with the recorded data used to both determine the developmental duration and calculate the adult eclosion rate. Sex was determined based on morphological differences in the pupal exuviae, and the detailed protocol for sex identification of pupae is provided in Supplementary Table S1. Newly emerged adults, developed from pupae previously exposed to each exogenous melatonin concentration, were paired at a 1:1 male-to-female ratio and transferred into insect rearing jars (the same containers as for rearings). A 10% (*w*/*v*) aqueous honey solution was supplied in each container as a nutrient source for adult feeding. Egg production and adult survival were recorded daily until all individuals had died. Fresh soybean pods (without exogenous melatonin treatment) were supplied regularly as oviposition substrates. Before the experiment, 30–50% of the total mating pairs per treatment group were reserved as backup males, which were sourced from the same treatment group and batch, sex-identified in advance via pupal exuviae morphology, and reared separately in same-sex cohorts for eclosion to avoid premating. When a male adult died, a healthy backup male with an eclosion age within 12 h of the dead individual was supplemented to maintain the mating pair. At the end of the experiment, fecundity-related parameters, including total egg production, pre-oviposition period, oviposition period, and adult female lifespan, were calculated. The egg hatching rate was also determined.

Five independent biological replicates were performed for all experimental endpoints in this study. For each replicate, only healthy and valid larvae, which were individually reared, free of mechanical damage and rearing disturbance, and had complete full-cycle developmental records, were used for all assays. For developmental and survival endpoints, including larval and pupal developmental duration, body length, body weight, pupation rate, adult eclosion rate, and larval survival rate, 10 qualified, valid larvae were included per melatonin treatment group per biological replicate. For reproductive parameters, including pre-oviposition period, oviposition period, female adult lifespan, and lifetime fecundity, 5 valid larvae reared under identical standardized rearing conditions were included per melatonin treatment group per biological replicate. The value of each endpoint was calculated based on valid samples within a single biological replicate, and all final statistical analyses were performed exclusively on the dataset derived from the five independent biological replicates.

### 2.3. Determination of Nutrient Content in Mature Larvae

Larval glycogen, total sugar, and protein contents were determined using mature fifth-instar *L. glycinivorella* larvae from different exogenous melatonin treatment groups. Three independent biological replicates were conducted for each treatment, with 5 healthy, developmentally synchronized larvae pooled per replicate. Three technical replicates were run for each pooled sample, and the mean of the technical replicates was taken as the final value of the corresponding biological replicate. All statistical analyses were performed exclusively based on data from the 5 independent biological replicates. Pre-experiments verified that this design met the basic statistical power requirements, and pooled sampling effectively mitigated interference from inter-individual physiological variation on the assay results. Fat content was calculated using processed mature *L. glycinivorella* larvae from different melatonin treatment groups (the same samples for body weight and length measurement in Section 2.2), with 5 biological replicates per treatment and 10 larvae per replicate.

Mature larvae from each melatonin treatment group were dried in an oven (LC-101-1B, Shanghai Lichen Bangxi Instrument Technology Co., Ltd., Shanghai, China) at 60 °C until a constant weight was reached, and their dry weight (DW) was measured and recorded. Individually weighed mature larvae were homogenized in a chloroform–methanol solution (chloroform:methanol, 2:1, *v*/*v*). The homogenate was centrifuged at 10,000× *g* for 10 min at 4 °C (model number: 5430R, Eppendorf (Shanghai) Laboratory Technology Co., Ltd., Shanghai, China), and the supernatant was discarded. This centrifugation step was repeated multiple times. The residue was dried to a constant weight to obtain the larval dry weight (LDW). The fat content of each larva was then calculated using Formula (1):

(1) $$\mathrm{Fat}\;\mathrm{conten}t\;=\;\frac{\left(\mathrm{DW}-\mathrm{LDW}\right)}{\mathrm{DW}}\;\times \;100$$

where *DW* is dry weight (g); *LDW* is the larval dry weight (g).

Glycogen, total sugar, and protein contents were determined using commercial assay kits supplied by Jiangsu Meibiao Biotechnology Co., Ltd. (Yancheng, China). The total sugar assay was performed at a wavelength of 500 nm, using the standard curve equation: *y* = 7.229*x* + 0.028 (R^2^ = 0.999). Protein content was measured at 562 nm with the standard curve: *y* = 0.180*x* + 0.018 (R^2^ = 0.99). All measurements were performed in triplicate.

Glycogen content was determined based on tissue sample mass (g), and the extraction buffer was added at a ratio of 1:10 (volume in mL per gram of tissue). The samples were then homogenized to obtain a uniform homogenate. Using a microplate reader (Labsystems Multiskan MS 352, Artisan Technology Group, Champaign, IL, USA) set to 510 nm, the required reagents were added sequentially, and the optical density (OD) of each sample was measured. Glycogen content was calculated from the OD values according to the kit protocol. The glycogen content was calculated using Formula (2):

(2) $$\mathrm{Glycogen}\;\mathrm{content}\;=\;0.9\;\times \;∆A_{\mathrm{glycogen}}\;÷\;(A_{\mathrm{standard}}-A_{\mathrm{blank}})\;÷\;W\;\times \;D$$

where *W* is the sampling quantity (g); *D* is the dilution factor of the sample before testing (*D* = 1 if undiluted).

### 2.4. Determination of Freezing Point and Supercooling Point

For *L. glycinivorella* used in all melatonin concentration gradient treatments, the insect source acquisition and standard rearing procedures were consistent with those detailed in Section 2.1 and Section 2.2. For each melatonin concentration treatment, mature larvae of *L. glycinivorella* that had been removed from pods and were 5 days old were selected for the experiment. The control group (CK) was held in an illuminated incubator at 21 °C until measurement. The mature larvae in the remaining melatonin treatment groups were separately subjected to cold acclimation at −10 °C for 4 h, followed by recovery in an illuminated incubator at 21 °C for 24 h. Mature larvae of *L. glycinivorella* treated with different concentrations of melatonin were individually placed into 1.5 mL centrifuge tubes. The thermistor probe of a supercooling point detector was placed in close contact with the larval body and fixed with cotton to avoid physical injury. Changes in larval body temperature were recorded using an insect supercooling point detector (model number: SUN-V, Beijing Pengcheng Electronic Technology Center, Beijing, China) connected to a computer, which generated a temperature–time curve. Based on this curve, the supercooling point and freezing point of each larva were determined. Five biological replicates were set for each melatonin treatment, with 5 healthy, developmentally synchronized larvae included per biological replicate.

### 2.5. Data Analysis

All statistical analyses in this study were performed using IBM SPSS Statistics 21.0 (IBM Corp., Armonk, NY, USA), with a two-tailed significance level set at α = 0.05.

Prior to formal statistical analysis, the normality of data distribution was jointly assessed via the Shapiro–Wilk test and Shapiro–Francia test, while homogeneity of variances across treatment groups was verified using Levene’s test (*p* > 0.05 indicated homogeneous variance). The appropriate statistical method was selected for each variable according to the results of normality and homogeneity of variance tests, as well as the data type.

For variables conforming to normal distribution with homogeneous variance (pupal duration, larval body weight, pupal body length, pupal body weight, pre-oviposition period, oviposition period, female adult lifespan, and total sugar content), data were presented as mean ± standard error (SE). Inter-group comparisons were conducted using one-way analysis of variance (ANOVA), followed by Tukey’s HSD post hoc test for multiple comparisons (Table S2). For variables with heterogeneous variance across groups (larval developmental duration and larval body length), pairwise comparisons were performed using Welch’s ANOVA coupled with the Games–Howell post hoc test.

For the discrete count data of eggs laid per female of *L. glycinivorella*, an overdispersion test was first conducted by establishing a Poisson regression model through the generalized linear model (GENLIN) module. The result showed that the ratio of the Pearson chi-square value to the degrees of freedom (*df*) was 404.246, which indicated extremely significant overdispersion of the data and a violation of the distribution assumption of Poisson regression. Therefore, a generalized linear model with a negative binomial distribution was ultimately adopted to analyze the overall differences among different melatonin treatment groups, and Bonferroni correction was applied to adjust the significance level for post hoc multiple comparisons.

For box plot visualization, the box represents the 25th to 75th percentiles (interquartile range, IQR), the solid line within the box indicates the median, and the whiskers extend to the extreme values within 1.5 times the IQR. In all analyses, different lowercase letters above boxes, bars, or within the same column denote statistically significant differences between treatment groups (*p* < 0.05). No significance letters were assigned to the total sugar content, as no statistically significant difference was detected among all treatment groups.

## 3. Results

### 3.1. Developmental Duration of L. glycinivorella

**Figure 1:**
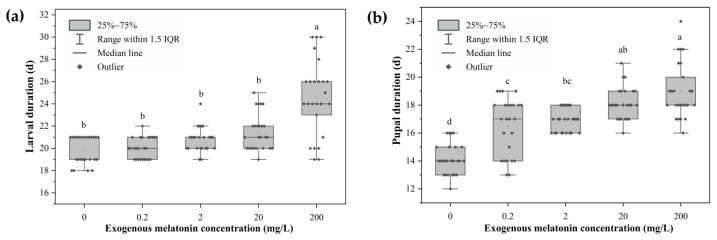
Larval duration and pupal duration of *L. glycinivorella* treated with different melatonin concentrations. (**a**) Larval developmental duration. (**b**) Pupal duration. Different lowercase letters above the boxes indicate significant differences among treatments at the *p* < 0.05 level. The box plots display the raw data distribution of 50 individuals, and statistical significance was determined based on the mean values of the 5 independent biological replicates.

The developmental durations of larvae and pupae exposed to different melatonin concentrations are shown in Figure 1. Melatonin treatment significantly affected both larval and pupal development, while developmental duration exhibited a non-significant increasing trend with rising melatonin concentrations. Different treatments exerted a highly significant effect on pupal duration (*F*_(4,20)_ = 38.048, *p* < 0.001). Pupal duration was significantly prolonged with increasing melatonin concentrations. The pupal duration in the untreated control group (0 mg/L melatonin) was significantly shorter than that in all other melatonin-treated groups, while no significant differences in pupal duration were detected among the 0.2, 2, 20, and 200 mg/L melatonin treatment groups (*p* > 0.05). Larval developmental duration was also strongly modulated by melatonin treatment, with significant regulatory effects detected across the tested concentrations (Welch’s ANOVA, *F*_(4,9.603)_ = 17.162, *p* < 0.001). There were no significant differences in larval developmental duration among the 0, 0.2, 2, and 20 mg/L melatonin groups (Games–Howell post hoc test, *p* > 0.05), whereas the larval developmental duration of the 200 mg/L melatonin group was significantly longer than that of all other groups (Games–Howell post hoc test, *p* < 0.05).

**Figure 2:**
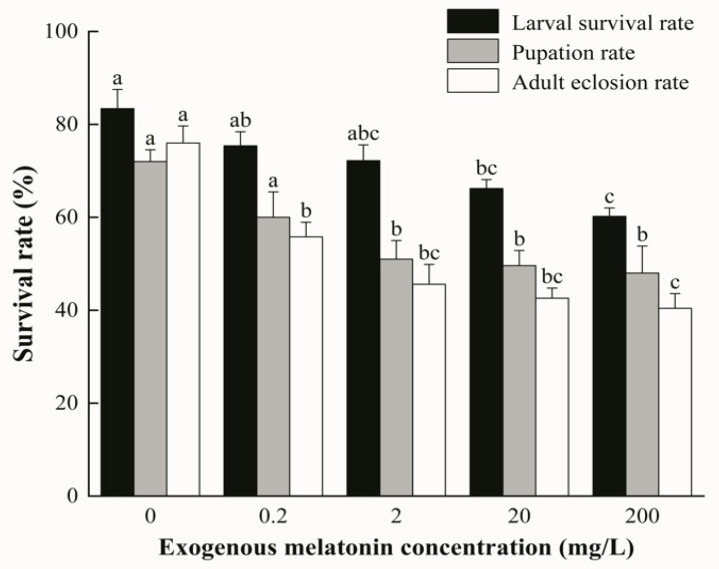
Larval survival rate, pupation rate, and adult eclosion rate of *L. glycinivorella* treated with different melatonin concentrations. Different lowercase letters indicate significant differences among treatments at *p* < 0.05.

Larval survival rate, pupation rate, and adult eclosion rate differed significantly among different melatonin treatments (Figure 2). Significant overall differences were detected among different melatonin treatments for larval survival rate (*F*_(4,20)_ = 8.728, *p* < 0.001), pupation rate (*F*_(4,20)_ = 18.927, *p* < 0.001), and adult eclosion rate (*F*_(4,20)_ = 5.170, *p* < 0.05). Pairwise comparisons showed that all three indicators in the control group (0 mg/L) were significantly higher than those in the 200 mg/L treatment group (all *p* < 0.05).

**Figure 3:**
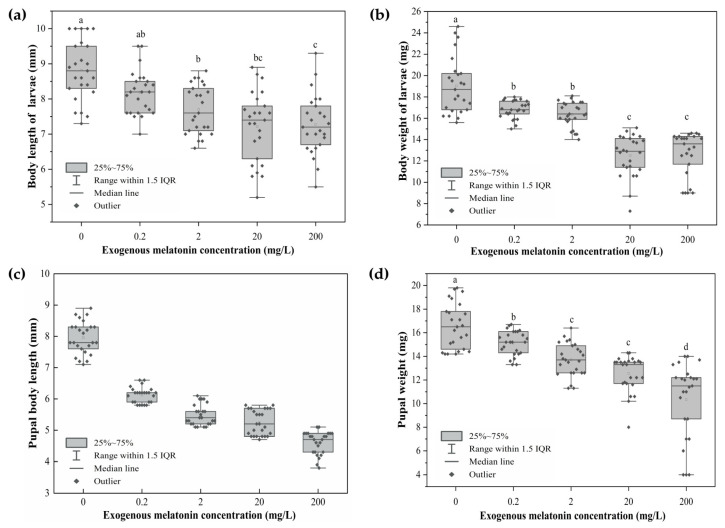
Body weight and length of *L. glycinivorella* larvae and pupae treated with different melatonin concentrations. (**a**) Larval body length, (**b**) larval body weight, (**c**) pupal body length, and (**d**) pupal weight. Different lowercase letters above the boxes indicate significant differences among treatments at the *p* < 0.05 level. The box plots display the raw data distribution of 50 individuals, with statistical significance determined based on the mean values of 5 independent biological replicates.

Significant differences were observed in the body weight (*F*_(4,20)_ = 90.278, *p* < 0.001) and body length (Welch’s ANOVA *F*_(4,9.794)_ *=* 22.225, *p* < 0.001) of pod-removed larvae among treatments, and both traits declined progressively with increasing melatonin concentration (Figure 3a,b). A similar trend was observed in pupae (Figure 3c,d). Exogenous melatonin exerted a highly significant effect on pupal weight (*F*_(4,20)_ = 67.600, *p* < 0.001) and pupal body length (*F*_(4,20)_ = 49.932, *p* < 0.001). In the control group (0 mg/L), larvae had the greatest body weight (19.04 mg) and a body length (8.82 mm). At 200 mg/L, body weight declined to 12.68 mg and body length to 7.28 mm. Pupal body weight and body length were highest in the control group, reaching 16.56 mg and 7.96 mm. At 200 mg/L, pupal body weight decreased to 10.32 mg and body length to 4.60 mm, which represented reductions of 6.24 mg and 3.36 mm compared with the control.

### 3.2. Nutrient Composition of L. glycinivorella

The effects of different melatonin concentrations on the nutrient composition of mature *L. glycinivorella* larvae are shown in Table 1. Exogenous melatonin exposure significantly modulated the accumulation of core energy reserve metabolites in the body, with robust regulatory effects on body glycogen content (*F*_(4,10)_ = 25.644, *p* < 0.001), body protein content (*F*_(4,10)_ = 16.959, *p* < 0.001), and body lipid content (*F*_(4,20)_ = 38.096, *p* < 0.001), while it did not induce a significant change in total body sugar content across the tested concentration gradients (*F*_(4,10)_ = 3.083, *p* = 0.068). The mean total sugar content was highest in the control group (0 mg/L), which was higher than that in the other four melatonin-treated groups. The maximum protein content was recorded in the control group (0 mg/L), which was significantly higher than that in all four melatonin-treated groups (*p* < 0.05), with no significant differences observed among the remaining treatment groups. The fat content of mature larvae differed significantly among melatonin treatments and declined as melatonin concentration increased. Fat content was highest under the control treatment (0 mg/L) and lowest at 200 mg/L (*p* < 0.05). In contrast, glycogen content increased with higher melatonin levels, rising from 45.12 mg/g in the control group to 48.91 mg/g at 200 mg/L (*p* < 0.05).

### 3.3. Reproductive Parameters of L. glycinivorella

The pre-oviposition period, oviposition period, fecundity, and female adult lifespan of *L. glycinivorella* all exhibited significant differences among melatonin treatments and declined as melatonin concentration increased (Table 2). Treatments with different concentrations of melatonin exerted a significant overall effect on the pre-oviposition period of *L. glycinivorella* (*F*_(4,20)_ = 5.268, *p* < 0.05) and highly significant overall effects on its oviposition period (*F*_(4,20)_ = 24.450, *p* < 0.001) and female adult longevity (*F*_(4,20)_ = 124.168, *p* < 0.001).

All reproductive parameters of *L. glycinivorella* decreased significantly with rising exogenous melatonin concentration, with the longest pre-oviposition period, oviposition period, and female adult longevity recorded in the 0 mg/L control group. The control group had a significantly longer pre-oviposition period than the 20 mg/L and 200 mg/L groups (*p* < 0.05), and a significantly longer oviposition period than all the melatonin-treated groups (*p* < 0.05), and the highest concentration group showed significantly shorter female longevity than all other groups (all *p* < 0.001).

A negative binomial generalized linear model (overall fit: likelihood ratio *χ*^2^ = 14.907, *df* = 4, *p* = 0.005) revealed that the *L. glycinivorella* oviposition amount decreased significantly with increasing melatonin concentration. The 0 mg/L control group had significantly higher oviposition than the 200 mg/L highest concentration group (*p* = 0.018), confirming that high-concentration melatonin significantly inhibited the oviposition capacity of this pest.

### 3.4. Supercooling and Freezing Point of L. glycinivorella

**Figure 4:**
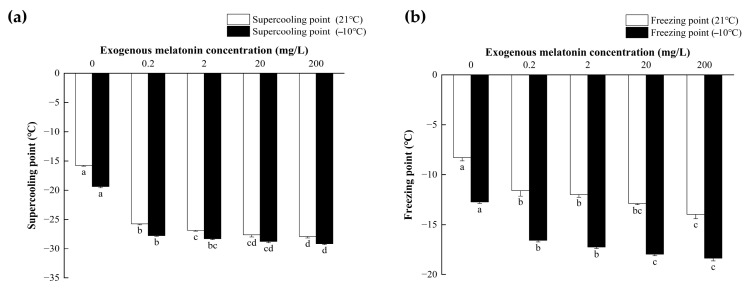
Effects of exogenous melatonin treatment on the supercooling point and freezing point of mature *L. glycinivorella* larvae. (**a**) Supercooling point and (**b**) freezing point. Different lowercase letters indicate significant differences among treatments at *p* < 0.05.

Melatonin treatment significantly enhanced the cold hardiness of mature *L. glycinivorella* larvae, as evidenced by marked alterations in two core cold tolerance indicators: supercooling point (SCP) and freezing point (FP). Across both 21 °C and −10 °C acclimation conditions, larval SCP and FP were highly significantly affected by melatonin concentration (SCP at −10 °C: *F*_(4,20)_ = 595.565, *p* < 0.001; FP at −10 °C: *F*_(4,20)_ = 182.437, *p* < 0.001; SCP at 21 °C: *F*_(4,20)_ = 467.711, *p* < 0.001; FP at 21 °C: *F*_(4,8.436)_ = 44.315, *p* < 0.001). For both indicators, post hoc pairwise comparisons confirmed that values in the 0 mg/L melatonin control group were significantly higher than those in all melatonin-treated groups (*p* < 0.05 for all comparisons), demonstrating that melatonin exposure significantly reduced larval SCP and FP (Figure 4).

## 4. Discussion

The results of the present study demonstrated that exogenous melatonin significantly prolonged the larval and pupal developmental duration of the *L. glycinivorella*; reduced the larval survival rate, pupation rate, and adult eclosion rate; and decreased the body weight of both larvae and pupae. These findings are consistent with previous observations that exogenous melatonin exerts detrimental effects on the growth and development of other insect species [32,34,39]. The inhibitory effect of exogenous melatonin on the growth and development of *L. glycinivorella* was also reflected in the aspect of nutritional accumulation. The total sugar, protein, and lipid contents of mature *L. glycinivorella* larvae exhibited a downward trend with increasing melatonin concentration. Nutritional accumulation during the larval stage directly determines the developmental performance and fecundity of insects [40]. In this study, we found that high concentrations of melatonin significantly reduced the fecundity of *L. glycinivorella*, which was in strong agreement with the observations of Zhang et al. [26] in the fall armyworm (*Spodoptera frugiperda*) and the report by Finocchiaro et al. [41] that exogenous melatonin reduced the mating frequency and oviposition output of fruit flies (*Drosophila melanogaster*). We hypothesize that the underlying mechanism may be that exogenous melatonin disrupts the normal processes of nutritional accumulation and energy metabolism in *L. glycinivorella* larvae, thereby impairing their growth, development, and reproductive capacity.

Upon herbivory infestation in soybean pods, a suite of defense mechanisms is induced, among which the accumulation of flavonoids is a pivotal component [42,43]. Flavonoids are secondary metabolites synthesized by plants, which play critical roles in plant responses to a wide range of environmental stresses (including both biotic and abiotic stresses) [44,45] and thereby enhance plant resistance to insect herbivores [46]. Previous studies have confirmed that exogenous melatonin can significantly elevate the contents of total phenols, total flavonoids, and various isoflavones in soybean cells [47,48,49,50]. It can also achieve the control of multiple agricultural pests, including *Aphis gossypii* on cucumber, mango gall midges, and *Sogatella furcifera* on rice, by inducing host plant resistance and modifying the feeding behavior of phytophagous insects [33,51,52]. We hypothesize that exogenous melatonin may alter the physiological status and chemical communication pathways of host soybean, thereby indirectly affecting the fitness of herbivorous pests. This hypothesis aligns with the core perspective proposed by Zhang et al. [53] under the framework of bottom-up effects in agroecosystems: plant-associated regulatory factors can mediate the trajectory of microbe–plant–insect multitrophic interactions by reshaping plant chemical signals and metabolic phenotypes and ultimately modulate the behavior and fitness of phytophagous pests [53]. In the present study, we also found that the total protein and lipid contents in mature larvae of the *L. glycinivorella* that fed on soybean pods were significantly reduced after the host plants were treated with exogenous melatonin. We speculate that exogenous melatonin treatment may significantly increase the content of flavonoid-based, insect-resistant secondary metabolites in soybean pods. These compounds can act as antifeedants to inhibit the feeding behavior of *L. glycinivorella*, resulting in insufficient nutrient intake and sustained starvation stress in the larvae, which in turn triggers the in vivo catabolic programs of lipid and protein [54,55,56].

In terms of the regulatory mechanism, the effects of melatonin on the *L. glycinivorella* may be closely associated with conserved pathways related to insect photoperiodic response and energy allocation. It is well established that insects can endogenously synthesize melatonin [57], and its biosynthetic process follows a circadian rhythm regulated by the light–dark cycle [58]. However, the direct mechanism by which melatonin modulates carbohydrate and lipid metabolism in *L. glycinivorella* remains unclear to date. Nevertheless, we observed in the present study that glycogen content increased significantly with increasing melatonin concentration (*p* < 0.05), which suggests a potential physiological adaptation strategy. As a key energy storage molecule in diapausing insects [59,60], *L. glycinivorella* may resist melatonin-induced stress and maintain its own energy homeostasis by regulating metabolic processes. This hypothesis is consistent with the findings of a study on the brown marmorated stink bug (*Halyomorpha halys*), in which melatonin regulated energy allocation under long-day conditions [32]. It also aligns with the well-documented role of glycogen as a precursor of cryoprotectants to enhance the cold hardiness of insects [61,62,63].

Under both 21 °C and −10 °C cold acclimation conditions, exogenous melatonin significantly reduced the supercooling point (SCP) and freezing point (FP) of *L. glycinivorella* (*p* < 0.05), indicating an enhancement in its low-temperature stress resistance. This may be attributed to the capacity of melatonin to maintain cellular redox homeostasis, alleviate oxidative damage induced by low temperature and other abiotic stressors [64,65], and upregulate the activities of antioxidant enzymes [66,67]. This mechanistic inference is corroborated by previous research documenting a similar melatonin-mediated reduction in SCP and FP in *Spodoptera frugiperda* [68]. Given that exogenous melatonin treatment decreases the SCP and FP of *L. glycinivorella* larvae and thereby enhances their overwintering cold tolerance, exogenous melatonin can be preferentially applied in regions with relatively mild winter temperatures, including the Huang–Huai River Valley main soybean-producing areas and southern soybean-producing regions of China. For the northern spring soybean-producing regions in China, the conventional agronomic practice of deep spring plowing can be implemented as a supporting mitigation measure. This practice can overturn overwintering cocoons from deep soil layers to the soil surface, disrupt the microhabitat required for larval diapause and overwintering, significantly elevate overwintering larval mortality, effectively suppress the initial population base at the source, and completely offset the potential ecological risks associated with exogenous melatonin application.

Future research should focus on elucidating the specific molecular mechanisms underlying the melatonin-associated physiological changes described above, with particular emphasis on the regulatory pathways through which melatonin modulates energy metabolism and circadian rhythms in soybean. Although melatonin has demonstrated promising application potential in integrated pest management (IPM) owing to its dual traits of disrupting insect growth and enhancing plant stress resistance [33,48,49], rigorous caution must be exercised with respect to its practical deployment for *L. glycinivorella* management. Prior to large-scale field application, further investigations are warranted to verify its actual control efficacy under natural field conditions. Meanwhile, the present study only assessed the effects of melatonin on larval cold tolerance physiology under controlled laboratory conditions, with no in situ field overwintering monitoring performed to date. Subsequent field trials will be conducted in accordance with pesticide efficacy trial guidelines to further evaluate the net effect of melatonin on larval overwintering survival, optimize compatible application protocols, and ensure the environmental safety of its field implementation [69].

## 5. Conclusions

Our findings demonstrate that exogenous melatonin mediates physiological and adaptive trait regulation in *L. glycinivorella*. High melatonin concentrations suppress key fitness traits associated with population growth while simultaneously enhancing cold tolerance in mature larvae by reducing their supercooling and freezing points. This dual regulatory effect advances our understanding of melatonin’s role in insect adaptive physiology and provides foundational insights for further research into melatonin-mediated pest population management.

## Figures and Tables

**Table 1:** Effects of melatonin concentrations on nutrients of mature *L. glycinivorella* larvae.

Exogenous Melatonin Concentration (mg/L)	Total Sugar Content (mg/g)	Glycogen Content (mg/g)	Protein Content (mg/g)	Fat Content (%)
0 (CK)	236.29 ± 12.47	45.12 ± 0.45 d	7.49 ± 0.19 a	51.31 ± 0.37 a
0.2	202.05 ± 6.61	46.13 ± 0.11 cd	6.89 ± 0.13 b	49.48 ± 0.26 b
2	201.68 ± 12.31	46.96 ± 0.28 bc	6.57 ± 0.16 bc	47.14 ± 0.41 bc
20	199.42 ± 12.69	47.79 ± 0.27 b	6.39 ± 0.07 bc	47.79 ± 0.59 c
200	185.84 ± 7.39	48.91 ± 0.24 a	6.11 ± 0.02 c	44.42 ± 0.38 d

Note: Total sugar content showed no significant difference among groups, with no significance labels added. Different lowercase letters indicate significant differences among treatments at *p* < 0.05. CK (control check): Blank control group treated with clean water containing 0 mg/L exogenous melatonin.

**Table 2:** Indicators of reproductive parameters of *L. glycinivorella* treated with different melatonin concentrations.

Melatonin Concentration (mg/L)	Pre-Oviposition Period (d)	Oviposition Period (d)	Fecundity (Egg)	Female Adult Lifespan (d)
0(CK)	6.10 ± 0.61 a	10.20 ± 1.18 a	145.83 ± 14.97 a	15.28 ± 0.14 a
0.2	5.00 ± 0.45 ab	7.70 ± 0.65 b	72.20 ± 15.09 bc	14.66 ± 0.49 ab
2	4.30 ± 0.37 ab	5.20 ± 0.33 c	52.80 ± 2.82 b	13.98 ± 0.29 b
20	3.70 ± 0.37 b	4.60 ± 0.43 c	45.00 ± 2.06 b	12.86 ± 0.27 c
200	3.50 ± 0.34 b	3.30 ± 0.47 c	30.10 ± 2.89 c	7.06 ± 0.16 d

Note: Different lowercase letters indicate significant differences among treatments at *p* < 0.05. CK (control check): Blank control group treated with clean water containing 0 mg/L exogenous melatonin.

## Data Availability

The raw data supporting the conclusions of this article will be made available by the authors on request.

## Supplementary Materials

The following supporting information can be downloaded at: https://www.mdpi.com/article/10.3390/biology15100750/s1. Table S1: Morphological diagnostic characteristics for distinguishing female and male pupae of *Leguminivora glycinivorella*. Table S2: Normality test, homoscedasticity test results, and statistical analysis strategy for all measured indices.
