# Supplementary material for: Effects of Exogenous Melatonin on Life History Traits and Cold Tolerance of Leguminivora glycinivorella (Lepidoptera: Tortricidae)
Source: Biology (Basel). 2026 May 9;15(10):750. doi: 10.3390/biology15100750 (PMC13203486; doi:10.3390/biology15100750)
Supplement: Supplementary file 1 [file biology-15-00750-s001.zip › biology-4265707-supplementary.pdf]

Supplementary Materials

Effects of Exogenous Melatonin on Life History Traits and Cold Tolerance of *Leguminivora glycinivorella* (Lepidoptera: Tortricidae)

Table S1 Morphological diagnostic characteristics for distinguishing female and male pupae of *Leguminivora glycinivorella*

Characteristic Dimension	Female Pupa	Male Pupa
Position of Gonopore	The gonopore (ovipore) is slit-shaped, located on the ventral side of the 8th abdominal segment, immediately adjacent to the posterior margin of the 7th abdominal segment, and forms a short longitudinal suture with the ovipore.	The gonopore is slit-shaped, located on the ventral side of the 9th abdominal segment, with a longer distance from the posterior margin of the 7th abdominal segment; a pair of small mammillary protrusions are usually present on both sides of the slit.
Ventral Morphology of Abdominal Segments	A distinct longitudinal suture is visible on the ventral side of the 8th abdominal segment; the ventral surfaces of the 8th and 9th abdominal segments are smooth overall, with no additional protruding structures.	No longitudinal suture is present on the ventral side of the 8th abdominal segment; the core diagnostic traits are concentrated on the 9th abdominal segment, with a more prominent gonopore structure.

Table S2. Normality test, homoscedasticity test results and statistical analysis strategy for all measured indices

No.	Measured Index	P value of Levene's Test	Normality Test Result	Homoscedasticity Test Result	Primary Statistical Method	Post-hoc Multiple Comparison Method
1	Pupal duration	0.089	Met	Met	One-way ANOVA	Tukey's HSD test
2	Larval survival rate	0.219	Met	Met	One-way ANOVA	Tukey's HSD test
3	Pupation rate	0.489	Met	Met	One-way ANOVA	Tukey's HSD test
4	Adult eclosion rate	0.602	Met	Met	One-way ANOVA	Tukey's HSD test
5	Pupal body length	0.549	Met	Met	One-way ANOVA	Tukey's HSD test
6	Pupal weight	0.784	Met	Met	One-way ANOVA	Tukey's HSD test
7	Larval body weight	0.406	Met	Met	One-way ANOVA	Tukey's HSD test
8	Total sugar content	0.682	Met	Met	One-way ANOVA	Tukey's HSD test
9	Glycogen content	0.116	Met	Met	One-way ANOVA	Tukey's HSD test
10	Protein content	0.115	Met	Met	One-way ANOVA	Tukey's HSD test
11	Fat content	0.126	Met	Met	One-way ANOVA	Tukey's HSD test
12	Pre-oviposition period	0.528	Met	Met	One-way ANOVA	Tukey's HSD test
13	Oviposition period	0.131	Met	Met	One-way ANOVA	Tukey's HSD test
14	Female adult lifespan	0.155	Met	Met	One-way ANOVA	Tukey's HSD test
15	SCP at 21 °C	0.079	Met	Met	One-way ANOVA	Tukey's HSD test
16	SCP after −10 °C cold acclimation	0.402	Met	Met	One-way ANOVA	Tukey's HSD test
17	FP after −10 °C cold acclimation	0.095	Met	Met	One-way ANOVA	Tukey's HSD test

18	FP at 21 °C	0.036	Met	Violated	Welch's ANOVA with Welch's correction	Games-Howe ll procedure
19	Larval development al duration	0.005	Met	Violated	Welch's ANOVA with Welch's correction	Games-Howe ll procedure
20	Larval body length	0.009	Met	Violated	Welch's ANOVA with Welch's correction	Games-Howe ll procedure
21	Fecundity	Not applicabl e	Violate d	Not applicable	Generalized Linear Model (GLM) with negative binomial distribution	Significance test based on GLM model output

Note: (1) Normality was verified via Shapiro-Wilk test, and homogeneity of variances was tested by Levene's test. A P-value > 0.05 indicates the data meets the assumptions of normality or homoscedasticity. (2) Abbreviations: ANOVA = Analysis of Variance; GLM = Generalized Linear Model; HSD = Honestly Significant Difference; SCP = Supercooling Point; FP = Freezing Point.
